# Influence of time between last myocardial infarction and prophylactic implantable defibrillator implant on device detections and therapies. *“Routine Practice” data from the SEARCH MI registry*

**DOI:** 10.1186/1471-2261-12-72

**Published:** 2012-09-11

**Authors:** Giuseppe Boriani, Gianluca Botto, Maurizio Lunati, Alessandro Proclemer, Boris Schmidt, Ali Erdogan, Werner Rauhe, Mauro Biffi, Elisabetta Santi, Daniel Becker, Marc Messier, Massimo Santini

**Affiliations:** 1 Institute of Cardiology, University of Bologna, Azienda Ospedaliera S. Orsola-Malpighi; Via Massarenti, 9 40138, Bologna, Italy; 2 Cardiology Department, Como, Italy; 3 Cardiology Department, Hospital, Niguarda, Milano, Italy; 4 Cardiology Department, Udine, Italy; 5 Cardiology Department, Freiburg Im Breisgau, Germany; 6 Cardiology Department, Giessen, Germany; 7 Cardiology Department, Bolzano, Italy; 8 Medtronic Italia, Roma, Italy; 9 Medtronic, Maastricht, Netherlands; 10 Cardiology Department, S.Filippo Neri Hospital, Roma, Italy

**Keywords:** Cardioverter defibrillator, Myocardial infarction, Registry, Sudden death, Ventricular tachyarrhythmias.

## Abstract

**Background:**

A multicenter European Registry, SEARCH-MI, was instituted in the year 2002 in order to assess patients’ outcomes and ICD interventions in patients with a previous MI and depressed LV function, treated with an ICD according to MADIT II results. In this analysis, we evaluate the influence of the time elapsed between last myocardial infarction (MI) and prophylactic cardioverter defibrillator (ICD) implant on device activations.

**Methods:**

643 patients with left ventricular dysfunction (mean LVEF 26 ± 5%) and NYHA class I-III were prospectively followed for 1.8 ± 1.2 years in a multicenter registry. The population was divided into 3 groups according to the time between last MI and ICD implant: [1] from 40 days to less than 1.5 years; [2] from 1.5 to less than 7 years and [3] at least 7 years.

**Results:**

The cumulative incidence of ventricular tachyarrhymias and appropriate device therapy (ATP or shock) were higher in patients implanted longer time from last MI (Gray’s Test p = 0.002 and p = 0.013 respectively). No significant differences were seen in all cause mortality (Gray’s Test p = 0.618) or sudden cardiac death across the MI stratification groups (Gray’s Test p = 0.663).

**Conclusions:**

Patients implanted with an ICD longer after the MI have a higher chance of presenting ventricular tachyarrhythmias and appropriate ICD therapy, while no differences were seen in overall mortality. These observations may be important for improving patient targeting in sudden death prevention.

## Background

The benefit of prophylactic ICD implant in appropriately selected patients with LV dysfunction has been demonstrated by prospective randomized clinical trials [1-5].

Nowadays there is increasing interest on two important issues: first, assessment of therapy in routine clinical practice [6,7] and second, identification of subgroups of patients with a higher chance of getting benefit from ICD therapy [8,9] or at the opposite with poor prognosis after myocardial infarction [10].

Since only 20-30% of patients implanted with an ICD for primary prevention of SCD present an appropriate intervention of the device in the first 3-5 years of follow-up, an analysis of the characteristics of patients who present an ICD intervention may provide interesting clinical implications, specifically with regard to improved patients’ targeting [2,3,8,9,11-14].

In patients with a previous MI associated with depressed left ventricular ejection fraction a post hoc analysis of the MADIT II trial (which enrolled patients between 1997 and 2001) showed that mortality rates in control patients increased as a function of time from most recent MI, with a greater survival benefit associated with ICD implant when the time elapsed from last MI was ≥18 months [13].

A multicenter European Registry, SEARCH-MI, was instituted in the year 2002 in order to assess patients’ outcomes and ICD interventions in patients with a previous MI and depressed LV function, treated with an ICD according to MADIT II results. The interim analysis of MADIT II-like patients enrolled in SEARCH MI registry was published in 2009 [11]. In this analysis, of the full cohort of patients enrolled in SEARCH MI we evaluate the influence of the time elapsed between last MI and prophylactic ICD implant on device activations.

## Methods

The SEARCH-MI registry was designed as a multi-centre, prospective, observational study, sponsored by Medtronic Inc. after publication of MADIT-II study [2]. The only inclusion criteria applicable to SEARCH MI registry were those related to the MADIT-II trial: all consecutive patients with previous MI one month or more before entry, LVEF lower or equal to 30%, without coronary revascularization within the preceding 3 months. Exclusion criteria were: implantation of ICD as secondary prevention of sudden death, age <18 years, unwillingness or inability to participate in data collection and any condition listed as class III in guidelines for defibrillator implantation. There was no upper age-limit.

The protocol was approved by the local ethics committees where required by national law and all patients gave their informed consent.

The patients enrolled underwent defibrillator implantation according to standard techniques: single-chamber, dual-chamber, or biventricular devices were implanted per treating physician prescription. All the devices were Medtronic Inc. market-released defibrillators. Device programming was empirical and programming of ATP in the fast VT zone according to PainFree study was recommended [15]. Follow-ups were performed accordingly to standard follow-up visit scheme of participating centres. No additional procedures beyond regular practice were required. Data on demographic and clinical characteristics (medical history, LVEF, NYHA class, QRS width, medications, arrhythmic history) were collected at baseline. At each follow-up examination, the following patient related data were collected: clinical status, NYHA class, heart failure and other-related hospitalizations, drug therapy changes, atrial fibrillation occurrence. The following device-derived data were also reported: ventricular arrhythmia documented by the ICD, ICD interventions, percentage of ventricular pacing.

The cause of death was provided by the attending physician or collected from clinical records or from interview of witnesses. Death was classified according to the following scheme: death occurring in the first hour from the onset of symptoms was defined as sudden death; death resulting from a cardiac event was defined as cardiac death. unwitnessed death was classified as sudden. To classify unreported mortality and patients lost to follow-up, patient’s information was retrieved from the regional demographic service.

Occurrence of ventricular tachyarrhythmias, ICD intervention, and other clinical data were prospectively collected. The time to the first appropriate treatment for ventricular arrhythmia was defined as time from implant to either intervention with anti-tachycardia pacing (ATP) or ICD shocks. Classification of spontaneous episodes and ICD therapies (both appropriate and inappropriate) stored in the device memory was adjudicated by a committee of five physicians in a blinded review process based on an internet platform (Web-EGM database). Each episode was independently reviewed by at least two physicians. In the case of disagreement, a third expert contributed to the final adjudication. Arrhythmic events were reported separately as appropriate detections and appropriate therapies to avoid bias from devices programmed in monitoring status.

The population was divided into the following 3 groups according to the time elapsed between last MI and prophylactic ICD implant: Group 1 (from more than 40 days to less than 1,5 years), Group 2 (from 1.5 years to less than 7 years) and Group 3 (at least 7 years after last MI). This grouping was based first on the median (2557 days, 7 years) and then given the results from the MADIT II post-hoc analysis [13], an additional cut-point corresponding to 1.5 years after last MI was also included.

### Statistical analysis

Descriptive statistics were used to describe the patient population. Comparisons between the baseline characteristics were performed by chi-square test for categorical variables and ANOVA for continuous variables. Cox regression models was used to assess the relationship between time from MI (as a continuous variable) and outcomes, results are reported as hazard ratios and 95% confidence intervals (CI). The cumulative incidences of outcomes from implant were calculated with all deaths included as competing outcomes and compared across the time since MI strata using Gray’s test [16]. In the calculation of cumulative incidence, only patients alive or lost at the end of follow up were censored. All probability values are two-tailed and a P-value < 0.05 was considered statistically significant. No adjustments to the p-values for multiple testing were made. All analyses were conducted using SAS 9.2, with the exception of the competing risk analysis that was conducted using R 2.13.1.

## Results

Seven hundred fifty seven patients were prospectively enrolled from 68 centres across 5 countries with the majority of patients enrolled from Italy (73%). For 114 patients the date of the MI was not known, since the MI had been silent. These patients have been excluded in the present analysis. Table 1 shows patient characteristics at baseline (in proximity to ICD implant date) in the cohort of 643 patients and in the 3 groups of patients stratified according to the time elapsed from last MI. Two hundred and two patients (31%) had a LVEF between 30% and 36%. A total of 182 arrhythmic events and 74 deaths were observed over a median follow up of 22 months (interquartile range,11 to 33 months). In Table 2 clinical outcomes and arrhythmic events by MI stratification group are reported. As shown, an higher occurrence of detected and appropriately treated (with ATP or shock) ventricular tachyarrhythmias was found in patients implanted with the ICD more than 7 years after last MI.

**Table 1:** Baseline patients’ characteristics in the cohort of 643 patients and in the 3 groups of patients stratified according to the time elapsed from last MI

	**All**	**Time from MI > 40 days and < 1,5 years**	**Time from MI ≥ 1,5 years and < 7 years**	**Time from MI ≥ 7 years**
	**N = 643**	**N = 158**	**N = 171**	**N = 314**
Age, years §				
Mean (±SD)	66.7 (±9.4)	66.4 (±11.1)	64.6 (±9.3)	67.9 (±8.4)
Male §	568 (88.3%)	129 (81.6%)	152 (88.9%)	287 (91.4%)
NYHA				
I	55 (8.6%)	12 (7.6%)	20 (11.8%)	23 (7.3%)
II	315 (49.3%)	73 (46.5%)	89 (52.7%)	153 (48.9%)
III	256 (40.1%)	65 (41.4%)	57 (33.7%)	134 (42.8%)
IV §	13 (2.0%)	7 (4.5%)	3 (1.8%)	3 (1.0%)
LVEF, %				
Mean (±SD)	25.9 (±5.0)	26.2 (±5.1)	25.6 (±5.0)	26.0 (±4.9)
Median (IQR)	26 (23, 30)	26 (24, 30)	26 (21, 30)	26 (23, 30)
History of permanent atrial fibrillation §	46 (7.2%)	8 (5.1%)	6 (3.5%)	32 (10.2%)
LBBB	168 (26.1%)	32 (20.3%)	46 (26.9%)	90 (28.7%)
Hypertension	301 (48.6%)	79 (52.0%)	85 (51.8%)	137 (45.2%)
Diabetes	182 (29.4%)	47 (30.9%)	45 (27.3%)	90 (29.7%)
CABG §	239 (37.2%)	33 (20.9%)	64 (37.4%)	142 (45.2%)
PTCA §	247 (38.4%)	86 (54.4%)	63 (36.8%)	98 (31.2%)
Amiodarone	154 (24.2%)	33 (21.2%)	48 (28.2%)	73 (23.5%)
Statins	319 (49.8%)	78 (49.7%)	91 (53.5%)	150 (47.9%)
Diuretics	545 (85.0%)	137 (86.7%)	140 (82.4%)	268 (85.6%)
Beta blockers	497 (77.8%)	128 (81.5%)	126 (74.1%)	243 (77.9%)
ACE inhibitors	507 (79.1%)	131 (82.9%)	129 (75.9%)	247 (78.9%)
Angiotensin II receptor inhibitors	52 (8.2%)	12 (7.7%)	13 (7.7%)	27 (8.7%)
Single-chamber ICD	341 (53.0%)	92 (58.2%)	87 (50.9%)	162 (51.6%)
Dual-chamber ICD	173 (26.9%)	36 (22.8%)	54 (31.6%)	83 (26.4%)
CRT-ICD	129 (20.1%)	30 (19.0%)	30 (17.5%)	69 (22.0%)
Time from last MI, years				
Median (IQR)	7.0 (1.6, 12.8)	0.5 (0.3, 0.9)	4.0 (2.7, 5.5)	12.9 (9.9, 17.4)

Numbers displayed as n (%), unless otherwise specified.

LVEF = Left Ventricular Ejection Fraction; LBBB = Left Bundle Branch Block; MI = Myocardial Infarction; IQR = Interquartile Range,SD = Standard Deviation

§ Significant difference (p < 0.05) at chi square test for proportions and ANOVA for means.

**Table 2:** Clinical outcomes and arrhythmic events in the 3 groups of patients, stratified according to the time elapsed from last MI

	**Time from MI > 40 days and < 1,5 years**	**Time from MI ≥ 1,5 years and < 7 years**	**Time from MI ≥ 7 years**
	**N = 158**	**N = 171**	**N = 314**
Follow-Up, months			
Median (IQR)	24 (15, 35)	22 (12, 34)	22 (11, 32)
Detection of at least 1 ventricular tachyarrhythmia (% ) §	33 (20.9%)	45 (26.3%)	104 (33.1%)
Delivery of at least 1 appropriate device therapy (ATP or shock) (%) §	27 (17.1%)	34 (19.9%)	82 (26.1%)
All cause mortality (%)	17 (10.8%)	19 (11.1%)	38 (12.1)
Cardiac mortality (%)	9 (5.7%)	8 (4.7%)	20 (6.4%)
SCD mortality (%)	3 (1.9% )	3 (1.8%)	9 (2.9%)

§ Significant difference between strata, p < 0,05.

SCD = sudden cardiac death.

**Figure 1:**
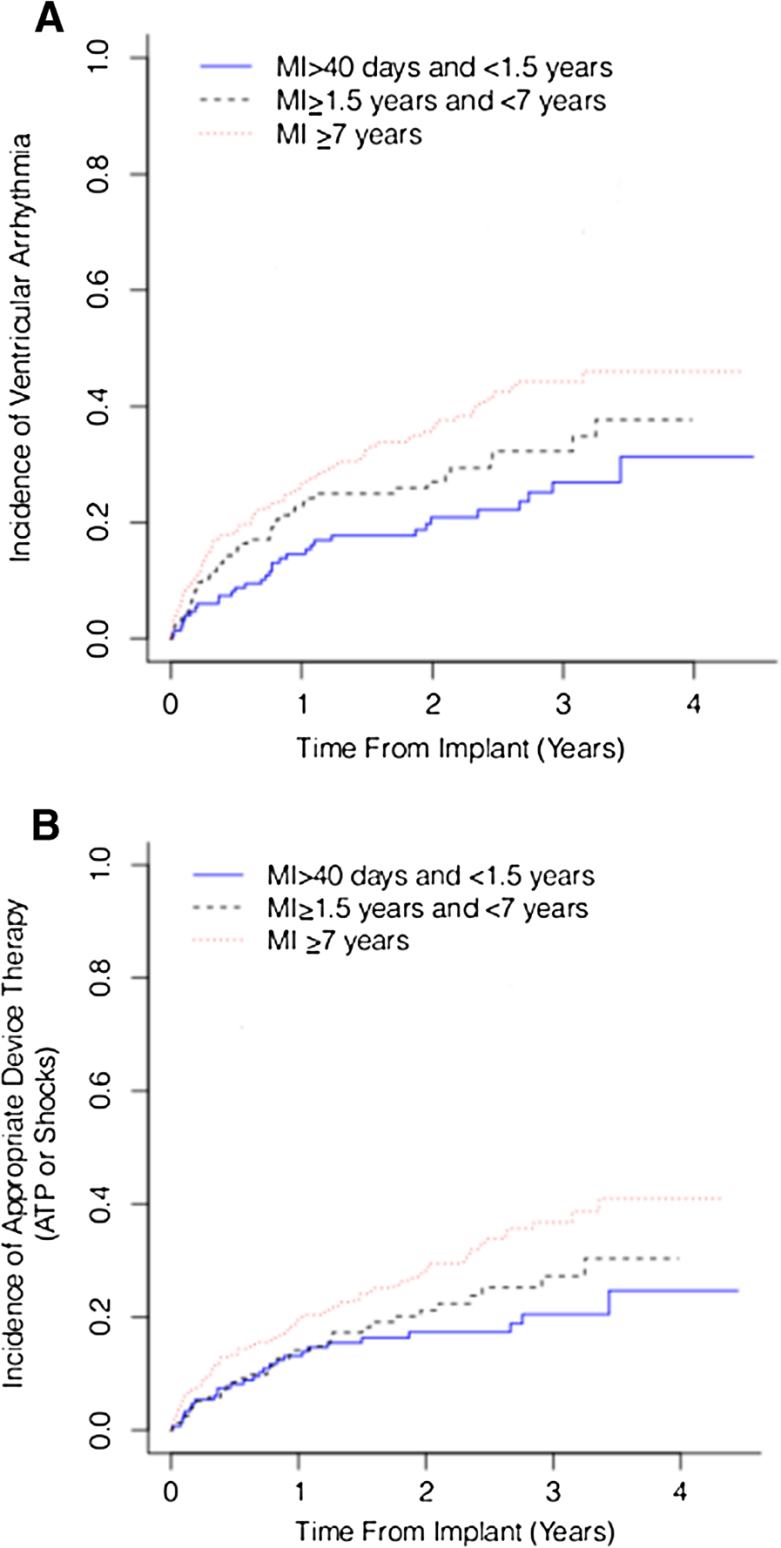
Cumulative incidence estimates of ventricular tachyarrhythmias (panel A) and appropriate device therapy (ATP or shock) (panel B) in patients with a prior MI ≥7years (dotted red line), MI ≥ 1.5 years and <7 years (dashed black line) and MI >40 days and <1.5 years (blue line).

**Figure 2:**
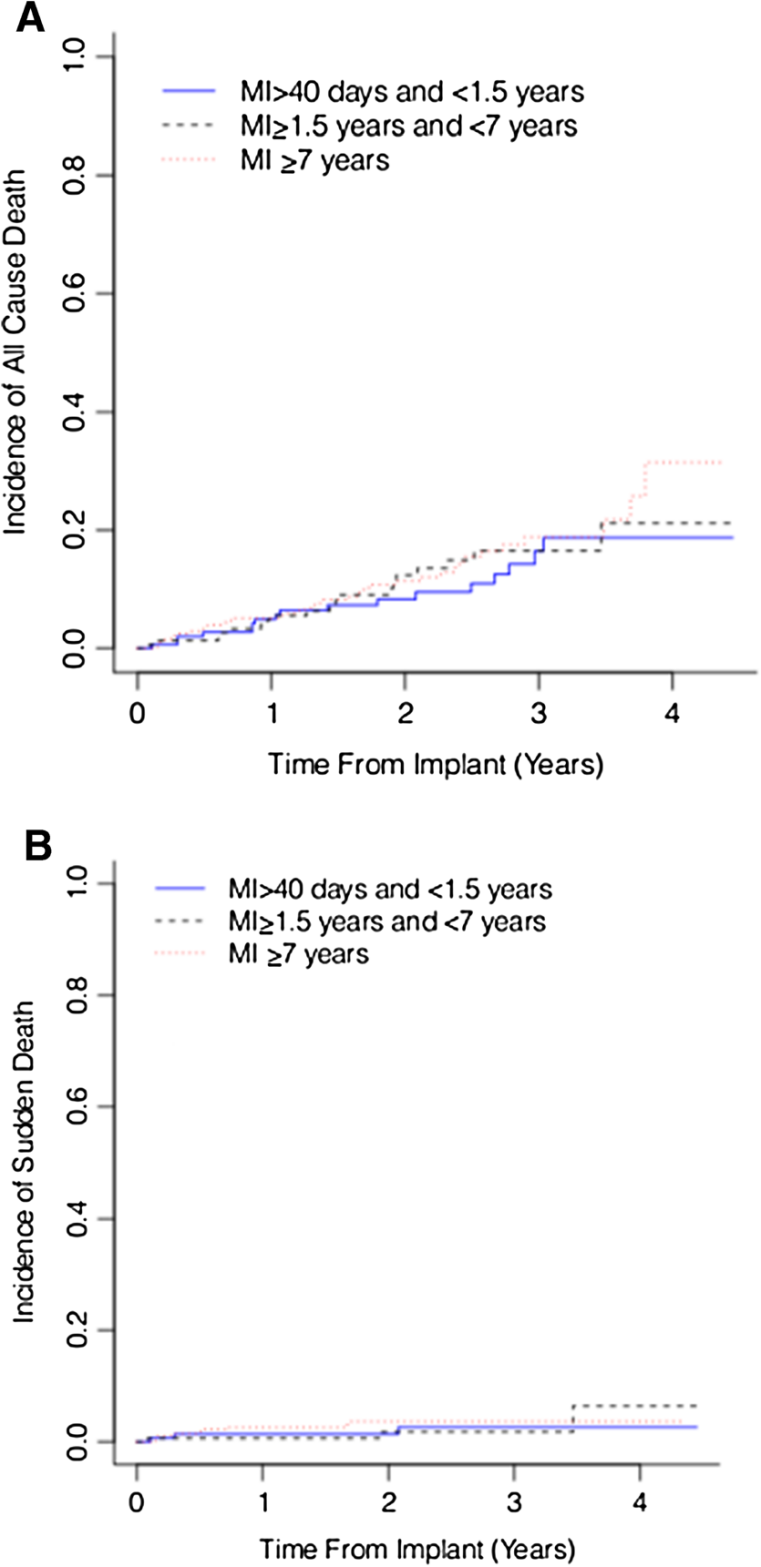
Cumulative incidence estimates of all cause mortality (panel A) and sudden cardiac death (panel B) in patients with a prior MI ≥7years (dotted red line), MI ≥ 1.5 years and <7 years (dashed black line) and MI >40 days and <1.5 years (blue line).

The cumulative incidence of ventricular tachyarrhythmias (Figure 1A) and appropriate device therapy (ATP or shock) (Figure 1B) were higher in patients with longer time elapsed from last MI (Gray’s Test p = 0.002 and p = 0.013 respectively). No significant differences were seen in all cause mortality (Figure 2A, p = 0.618) or sudden cardiac death (Figure 2B) across the MI stratification groups (Gray’s Test p = 0.663).

In a univariate Cox regression analysis of time from MI (continuous variable = years from MI) yielded a hazard ratio of 1.020 (95% CI 1.003-1.0388), p = 0.024 for detection of ventricular tachyarrhythmia and of 1.023 (95% CI 1.003-1.044), p = 0.0213 for appropriate device therapy (ATP or shock). After adjustment for age, NYHA, permanent AF status at baseline and implanted device type, time from MI had a hazard ratio of 1.021 (95% CI 1.002, 1.039), p = 0.026 for detection of ventricular tachyarrhythmia and 1.023, (95% CI 1.002, 1.044), = 0.029 for appropriate device therapy (ATP or shock). For all cause death and sudden cardiac death the hazard ratio was not statistically significant (adjusted hazard ratios 1.018 (95% CI 0.990, 1.046) and 1.016 (95% CI 0.958, 1.078) , respectively).

## Discussion

Despite the improvement in treatment of coronary artery disease and myocardial infarction, survivors of a previous myocardial infarction are exposed to substantial risk of life-threatening ventricular tachyarrhythmias which has been the basis for use of ICD in post-infarction patients with left ventricular dysfunction [4,5,17,18].

There is a need to evaluate in routine practice, the outcome of patients with a previous MI, implanted with an ICD for primary prevention of sudden death. This is in accordance to the current approach proposed for a comprehensive validation of effective impact of innovative therapies, based on combination of high quality clinical trials and prospective registries [6]. Either the potentially worse outcome of patients with a profile similar to that of controlled trials but not selected according to trial’s exclusion criteria [7], or the need to verify how treatments affect patient’s outcomes when applied in an unselected context, are at the basis of current interest on prospective registries [6].

Our study, which is based on the final results of a prospective multicenter registry [11], suggests that patients implanted with an ICD with remote myocardial infarction (7 years or more from last infarction) have higher chance of presenting an appropriate ICD activation, but do not present a worse outcome in terms of survival in comparison with post infarction patients who carry an ICD, but have a shorter time elapsed between last myocardial infarction and ICD implant. According to these data, the subgroup of patients with remote MI appears to include those patients who could get the greatest benefit from implant of a prophylactic ICD. However, these considerations should take into account that appropriate ICD shocks may occur more frequently than sudden death, as shown in patients with nonischemic cardiomyopathy [19] and therefore some limitations exists in use of appropriate ICD shocks as a surrogate of sudden cardiac death. An increase in appropriate shocks with increasing time after MI has also been reported for the patients with previous MI enrolled in the SCD-HeFT (Sudden Cardiac Death in Heart Failure Trial) study [20].

In the context of use of ICDs, there is growing interest on determinants of appropriate ICD therapy in patients with reduced ventricular function after myocardial infarction. This as a way for assessing what patients may benefit most from implantation of a prophylactic ICD. This kind of analysis, combined with analysis of relationships between determinants of ICD therapy and death may be the basis for any attempts to improve patients targeting. As a matter of fact, in the “routine practice” it is possible that competing causes of death due to heart failure, comorbidities or any other cause when occurring without any prior appropriate ICD intervention, may preclude benefit from ICD therapy [8].

Previously a post hoc analysis of the original MADIT II trial did not report any relationship between time from last MI and ICD activations, but showed that mortality rates in control patients increased as a function of time elapsed from most recent MI, with greater survival benefit associated with ICD implant when time elapsed from last MI was ≥18 months [14].

Recently a post-hoc analysis of MUSTT trial found that the risk of 2- and 5-year arrhythmic death, cardiac arrest, and all-cause death did not vary as a function of time from the last recent MI [21]. However, interpretation of these data should consider that the MUSTT was non-randomized and that the published analysis was based only on patients who were not treated with an ICD and who were discharged without antiarrhythmic medications.

In our study we found that the rate of ventricular tachyarrhythmias and appropriately delivered ICD therapies increase significantly with the time between prior MI and ICD implant. This suggests that for high risk patients the longer the time elapsed from last MI the higher is the tendency to spontaneously develop ventricular tachyarrhythmias. Patients with a long survival after MI appear to have a higher probability of ventricular tachyarrhythmias (and ICD intervention) probably related to an extensive remodeling, more pronounced than in patients with a recent MI. The relationship between time from myocardial necrosis and arrhythmogenesis is confirmed by findings of the Maastricht study on out-of-hospital cardiac arrest in the 1990's [22], where the mean interval between previous MI and occurrence of cardiac arrest was relatively long, being in average 6.5 years.

In daily clinical practice the question concerning what the optimal time would be for implanting an ICD in patients with previous MI and evidence of LV dysfunction is still open, and has public health implications. A recent modeling study indicates that benefits of ICD implantation in a relatively early phase after MI (ie, at 60 days) are really modest, when projected at 10 years of follow up, in comparison to delayed implantation at 6 months or 1 year [23]. Current practice guidelines recommend ICD implant in patients with previous MI and left ventricular dysfunction after at least 40 days from last MI, although the optimal time after the first 40 days remains undefined. It is unlikely that this question will be answered with a randomized controlled trial, therefore data from observational studies and registries play an important role. However we have to be cautious when interpreting the results because of possible selection bias and confounding factors.

Our registry, while limited to devices made by one manufacturer, included both single and dual chamber ICDs and devices with cardiac resynchronization therapy, a very effective therapeutic resource according to a series of controlled trials [24]. According to MADIT CRT trial cardiac resynchronization therapy will find a wider application in post infarction patients, since NYHA I-II patients will be candidate to resynchronization in the presence of wide QRS interval [25]. The MADIT CRT trial showed that devices with both defibrillation capabilities and cardiac resynchronization therapy have additional benefit with regard to heart failure hospitalization in comparison with ICD only therapy, but without advantages in terms of survival [25].

Most recently a subanalysis of the MADIT-CRT [26] demonstrated that among post MI patients the risk of life threatening ventricular arrhythmias increases as a function of time elapsed since revascularization procedure. With this regard we did not perform an analysis of the effects of revascularization and residual ischemia on ventricular tachyarrhythmias, ICD discharges and outcomes, since this is quite problematic in the setting of a registry. We cannot exclude that observed differences may be due to confounding factors that were not collected, including the number of previous MIs, the extent of myocardial scar, the exact time of revascularization and the evolution of revascularization practices and MI treatment over time (as suggested by the data in Table 1, patients with a more remote MI were treated differently from those with a more recent MI, with regard to use of CABG or PTCA). However, we need to consider that it is difficult in daily practice to obtain detailed information on previous revascularization procedures as the basis for individualized decision making. Similarly to MADIT II analysis [13] we did not considered all the events corresponding to a MI, but we considered only most recent MI and this may be a limitation of the analysis.

Similarly to other registries we have to consider a series of limitations typical of study design. Although device programming according to PainFree study [15] was suggested, differences in detection and therapy programming among the patient population could have influenced the results. Postmortem ICD interrogations were not recorded.

## Conclusions

Patients implanted with an ICD longer after the MI have a higher chance of presenting ventricular tachyarrhythmias and appropriate ICD therapy, while no differences were seen in overall mortality. These observations may be important for improving patient targeting in sudden death prevention.

## Competing interests

ES is an employee of Medtronic Italia, Roma, Italy and DB and MM employees of Medtronic Inc., Maastricht, Netherlands.

## Authors’ contributions

GBor, GBot, ML, AP, MS, conceived the study, and participated in its design and coordination and helped to draft the manuscript. DB and MM performed the statistical analysis and contributed to report of study results. BS, AE, WR, MB, ES helped to draft the manuscript. All authors read and approved the final manuscript.

## Pre-publication history

The pre-publication history for this paper can be accessed here:

http://www.biomedcentral.com/1471-2261/12/72/prepub
